# Progress on the mechanism of Polycystin-1 in bone remodeling

**DOI:** 10.3389/fcell.2025.1693704

**Published:** 2026-01-12

**Authors:** JinShi Guo, GuangXuan Hu, XiaoYing Li, XueJie Yi, Bo Chang, Tao Li

**Affiliations:** 1 School of Exercise and Health, Shenyang Sport University, Shenyang, Liaoning, China; 2 College of Physical Education, Liaoning Normal University, Dalian, China; 3 School of Sports Science, Zhuhai College of Science and Technology, Zhuhai, China; 4 Laboratory Management Center, Shenyang Sport University, Shenyang, Liaoning, China

**Keywords:** polycystin-1, transmembrane protein, bone remodeling, mechanical force, signaling

## Abstract

Polycystin-1 (PC-1), a transmembrane protein expressed on cell membranes, plays a vital role in cell signaling and intercellular adhesion. Existing studies have shown that PC-1 plays a pivotal role in bone remodeling and that PC-1 deficiency results in disrupted bone remodeling, which markedly affects bone mass and skeletal development. This review describes the molecular structure and biological function of PC-1 and analyzes the mechanism by which it maintains bone homeostasis and regulates osteoblast and osteoclast activity. Particular emphasis is placed on the role of PC-1 in mechanical force-triggered bone remodeling and its interaction with the transcriptional co-activator tafazzin. Moreover, this review outlines the potential applications of PC-1 in treating skeletal diseases, such as osteoporosis, fractures, and premature closure of cranial sutures, thereby providing a theoretical basis for future research.

## 1 Introduction

Bone remodeling is a pivotal physiological process that preserves the dynamic homeostasis of the skeletal system. This process entails meticulous synchronization between osteoclast-mediated bone resorption and osteoblast-mediated bone formation (Zhang et al., 2021). This equilibrium is crucial for sustaining bone homeostasis, facilitating microdamage repair, and enabling adaptation to mechanical loading (Xu et al., 2023). Polycystin-1 (PC-1) has emerged as a regulator of bone remodeling and has garnered attention due to its distinctive role in osteoblast mechanoreception and signal transduction (Huang et al., 2024).

PC-1 is a large transmembrane protein encoded by the polycystic kidney disease 1 gene (*PKD1*). The protein’s complex structural domain composition confers unique functional properties (Luo et al., 2023). The extracellular region contains multiple functional structural domains that enable it to sense environmental changes and mechanical stimuli (Weston et al., 2003; Nigro and Boletta, 2021). The transmembrane structures and intracellular regions are involved in signal transduction (Shillingford et al., 2006; Li, 2013; Xiao et al., 2023). This structure enables PC-1 to play several roles in bone remodeling.

Beyond its roles in bone biology, PC-1 dysfunction is implicated in multiple pathological conditions, highlighting its systemic importance in mechano-sensing and signaling regulation. In autosomal dominant polycystic kidney disease (ADPKD), *Pkd1* mutations cause PC-1 deficiency, leading to impaired mechanically induced Ca^2+^ influx, elevated cAMP levels, and PKA activation, thereby promoting tubular cell proliferation and cyst fluid secretion (Mangoo-Karim et al., 1989a; Mangoo-Karim et al., 1989b). Simultaneously, activation of ERK/MAPK, mTOR, YAP/TAZ, and STAT3/6 pathways disrupts cell polarity and induces metabolic reprogramming, driving cystogenesis (Boletta and Caplan, 2025; Luo et al., 2023; Belibi et al., 2011). These findings underscore PC-1 as a central mechano-sensor, whose dysregulation not only initiates cyst formation but may also influence tumorigenic signaling in other tissues. In cancer, PC-1 exhibits tissue-specific effects: in renal cell carcinoma, its loss activates mTOR/STAT3 signaling to create a pro-proliferative microenvironment (Gargalionis et al., 2020; Papavassiliou et al., 2019); in colorectal cancer, PC-1 acts in a context-dependent manner (Gargalionis et al., 2018a); whereas in breast, prostate, lung, ovarian cancers, and gliomas, PC-1 mainly exerts tumor-suppressive functions by inhibiting JAK2/STAT3, mTOR, FAK/Src, and YAP/TAZ pathways, thereby suppressing proliferation, migration, and epithelial–mesenchymal transition (EMT) while enhancing apoptosis (Papavassiliou et al., 2019; He et al., 2021; Gargalionis et al., 2019; Zoi et al., 2022). Given its diverse regulatory roles in proliferation and mechanotransduction, similar mechanisms may underlie the cardiovascular abnormalities observed in PC-1 deficiency. In the cardiovascular system, PC-1 regulates Ca^2+^ homeostasis, mechanosensation, and downstream signaling to maintain vascular tone, endothelial nitric oxide production, and myocardial contractility. Its deficiency leads to endothelial dysfunction, vascular remodeling, myocardial Ca^2+^ imbalance, and fibrosis, contributing to hypertension, cardiac hypertrophy, arrhythmia, and arterial fragility (Zoi et al., 2022; Altamirano et al., 2019). Interestingly, alterations in PC-1 expression have also been reported in certain inflammatory and dermatologic disorders, suggesting that its dysregulation extends beyond internal organs. The decreased expression of PC-1 at psoriatic lesions may activate the ERK/MAPK–mTOR axis, promoting keratinocyte proliferation, migration, and inflammatory responses, while impaired mechanosensory function may be associated with the Koebner phenomenon (Altamirano et al., 2019; Gargalionis et al., 2018b). Together, these pathological manifestations emphasize the pleiotropic roles of PC-1 across multiple tissues.

Therefore, this review examines the dual regulatory roles of PC-1 in bone remodeling. PC-1 maintains bone mass by promoting osteoblast differentiation and inhibiting the differentiation of bone marrow mesenchymal stem cells (BMSCs) into adipocytes (Puri et al., 2004). In addition, PC-1 has been identified as a regulator of osteoclast formation and activity (Huang et al., 2024), thereby providing a new perspective on the balance between bone remodeling and the complexity of PC-1 in regulating bone mass.

Osteoblasts are the most abundant cell type in bone tissue and are uniquely equipped to sense and convert mechanical signals from bone tissue (Uda et al., 2017). However, the precise mechanisms of mechanotransduction remain unclear. Besides known mechanosensitive proteins, such as piezo-type mechanosensitive ion channel component 1 (PIEZO1) and integrins (Zhou et al., 2024; Yang et al., 2024), PC-1 has been recognized as an important novel mechanoreceptor of mechanical force (Huang et al., 2024). In response to mechanical force stimulation, PC-1 activates multiple downstream signaling pathways, including calcineurin (CaN)/nuclear factor of activated T cells (NFAT), Janus tyrosine kinase (JAK)/signal transducer and activator of transcription (STAT), and protein kinase B (AKT)/β-catenin. These pathways regulate gene expression and function in osteoblasts, playing a pivotal role in bone remodeling (Dalagiorgou et al., 2013; Dalagiorgou et al., 2017; Wang et al., 2014). Concurrently, PC-1 is also implicated in the pathogenesis of skeletal diseases such as osteoporosis, fractures, and premature closure of the cranial suture (Liu et al., 2024; Katsianou et al., 2021).

Thus, this review analyzes the multifaceted roles of PC-1 in bone remodeling. In addition to deepening our understanding of the mechanisms of bone remodeling, this study also offers a theoretical basis for developing novel PC-1-targeting therapeutic strategies for skeletal disorders, such as osteoporosis, fractures, and premature closure of the cranial suture. Furthermore, this review reveals new avenues for the prevention and treatment of skeletal diseases.

## 2 Structure and function of polycystins

### 2.1 Molecular structure of polycystin

The polycystin family of proteins is primarily constituted of PC-1 and PC-2. PC-1 is a complex transmembrane protein (Hughes et al., 1995; Harris et al., 1995) primarily expressed in epithelial, endocrine, cardiomyocyte, skeletal muscle, and bone cells (Pedrozo et al., 2015; Qin et al., 2020). The protein’s molecular weight exceeds 460 kDa (Harris et al., 1995), and it has 11 transmembrane domains (Nims et al., 2003).

**FIGURE 1:**
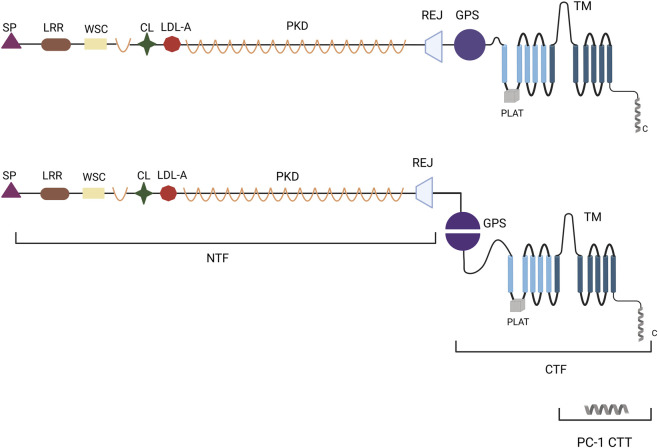
Schematic diagram of the structure of PC-1. LRR, leucine repeat sequence; Rej, receptor for egg jelly; LDL-A, low-density lipoprotein structural domain A; PKD, immunoglobulin-like repeats; WSC, cell wall integrity and stress component; GPS, G-protein-coupled receptor protein hydrolysis site; CL, C-type Lectin Domain; PLAT, Polycystin-1 Lipoxygenase Alpha-Toxin; TM, Transmembrane Domain; SP, Signal Peptide; PC-1 CTT, Polycystin-1 C C-Terminal Tail; NTF, N-Terminal Fragment; CTF, C-Terminal Fragment.

The extracellular region of PC-1 is complex and distinctive, comprising multiple functional structural domains. These include the leucine repeat sequence (LRR), receptor for egg jelly (Rej) region, low-density lipoprotein (LDL) structural domain A, immunoglobulin-like repeats, cell wall integrity and stress component, and the G-protein-coupled receptor protein hydrolysis site (GPS) at the base of the extracellular domain (Weston et al., 2003; Nigro and Boletta, 2021). Furthermore, PC-1 undergoes post-translational modification *via* GPS motifs near the membrane (Yu et al., 2007; Qian et al., 2002). This process results in the production of two fragments, an N-terminal fragment and a C-terminal fragment (CTF) (Qian et al., 2002; Wei et al., 2007). The extracellular N-terminal structural domain of PC-1 encompasses 3,048 amino acids and includes the LRR, C-type lectin, LDL-associated PKD repeats, and Rej structural domains (Stillman, 1994; Luo et al., 2023). In contrast, the C-terminus of PC-1 comprises 11 transmembrane and intracellular helical coil structural domains (Stillman, 1994; Luo et al., 2023). This region produces a C-terminal fragment (CTT) of approximately 30–35 kDa in size, which accumulates in the nucleus (Figure 1). PC-2, encoded by the polycystic kidney disease 2 (*PKD2*) gene, is a transient receptor potential channel comprising six transmembrane structural domains, an EF-chiral domain, and a coiled-coil structural domain at the C-terminus (Mochizuki et al., 1996).

### 2.2 Biological functions of polycystins

The molecular structure of PC-1 endows it with critical mechanosensory and signal transduction functions (Peintner et al., 2021; Amaral et al., 2022). Its extracellular PKD Ig-like domain exhibits remarkable extensibility and dynamic behavior under mechanical stress, enabling the recognition and transduction of mechanical signals through unfolding and refolding processes (Forman et al., 2005; Qian et al., 2005). Consequently, the N-terminal extracellular region is regarded as the “mechanosensing interface” of the cell, capable of converting external mechanical forces into intracellularly recognizable molecular signals, thereby activating downstream signaling pathways. Meanwhile, the C-terminal tail (CTT) is prone to cleavage under mechanical stimulation, translocates into the nucleus, and activates signaling cascades such as CaN/NFAT, JAK/STAT, and AKT/β-catenin, promoting the expression of osteogenic-specific factors including Runt-related transcription factor 2 (RUNX2), which accelerates osteoblast maturation and differentiation, regulates bone formation and accumulation, and is crucial for maintaining skeletal health (Dalagiorgou et al., 2013; Dalagiorgou et al., 2017; Wang et al., 2014).

PC-1 also interacts specifically with the extracellular matrix (ECM) through multiple domains, and the interplay between these domains determines its multifunctionality within the microenvironment. Studies have shown that the CL region significantly enhances ECM binding in the presence of Ca^2+^ (Weston et al., 2001), whereas the LRR region can bind matrix proteins and exert an inhibitory effect on cell proliferation (Malhas et al., 2002; Padhy et al., 2024). This indicates that PC-1’s interaction with the ECM is not a simple adhesion process but a regulated molecular recognition mechanism. Through differential domain interactions, PC-1 can modulate the distribution of cellular tension and integrate signaling, thereby influencing cell proliferation, migration, and differentiation.

Notably, PC-1 forms a complex with PC-2 and cooperates in multiple physiological processes (Wang et al., 2019). Su et al. elucidated the interaction mechanism between PC-1 and PC-2 through cryo-electron microscopy (CryoEM) (Su et al., 2018). Moreover, PC-1 and PC-2 form a heterotetrameric complex in a 1:3 ratio, with the transmembrane structural domain of PC-1 CTF binding to the transmembrane structural domain of PC-2 (Su et al., 2018). This complex serves as a mechanosensor that regulates bone mass (Katsianou et al., 2018). Nevertheless, there is debate among the scientific community regarding the ability of PC-1 and PC-2 to form nonselective cation channels. Co-expression of PC-1 and PC-2 at the cell membrane contributes to the formation of such channels (Delmas et al., 2004). In addition, PC-1 can independently form channels, even in the absence of PC-2 (Babich et al., 2004). Consequently, further in-depth studies are required to elucidate the specific roles of PC-1 and PC-2 in the channel formation process and the mechanisms of their interactions.

Notably, PC-2 deficiency impedes bone formation by osteoblasts, leading to low-conversion osteoporosis. This results in decreased bone density and disrupted bone microarchitecture, thereby increasing the risk of fracture (Xiao et al., 2014). In addition, *PKD2* deficiency reduces bone marrow adipogenesis and decreases the expression of peroxisome proliferator-activated receptor gamma (PPARγ), a key gene for adipocyte differentiation. This reduces the accumulation of bone marrow fat (Xiao et al., 2014). This finding contrasts with the role of PC-1 in bone marrow adipocytogenesis, suggesting that PC-1 and PC-2 have different effects on bone marrow adipogenesis. This further implies that they may have different signaling mechanisms in regulating bone remodeling (Xiao et al., 2014; Xiao et al., 2023).

Most research in this field has focused on PC-1, with relatively little attention directed toward PC-2. This is most likely because PC-2 primarily functions as a calcium channel involved in regulating intracellular calcium ion concentration rather than being directly involved in the response of osteoblasts to mechanical loading. Although PC-1 and PC-2 can form a complex to function together, existing studies suggest that PC-1 plays a more direct role in mechanosensing and bone density regulation, whereas PC-2 may serve an auxiliary or secondary role in these processes. Accordingly, this study focused on the impact of PC-1 on bone.

## 3 Role of PC-1 in bone remodeling

Bone remodeling is a process that maintains the integrity and mineral homeostasis of the adult skeleton. This process requires the synergistic action of multiple cells, including stem cell differentiation, osteoclast-mediated bone resorption, and osteoblast-mediated bone formation (Raggatt and Partridge, 2010; Hadjidakis and Androulakis, 2006). PC-1 deficiency results in a considerable reduction in bone density, impaired bone microarchitecture, and affects the mineralization process of the skeleton. This ultimately reduces bone mineral density (BMD) and results in a fragile skeleton (Xiao et al., 2023; Huang et al., 2024). Therefore, understanding the role of PC-1 in bone remodeling and its underlying mechanisms is important.

### 3.1 Effects of PC-1 on BMSC

During bone remodeling, PC-1 proteins play a dual role in promoting the differentiation and proliferation of osteoblasts and inhibiting adipocytosis in BMSCs (Xiao et al., 2018; Xiao et al., 2010). Specifically, PC-1 markedly reduces fat accumulation in the bone marrow by regulating the PPARγ signaling pathway in BMSCs. However, when the PC-1 protein loses its function, it exacerbates adipocytogenesis, leading to increased accumulation of adipose tissue (marrow adipose tissue) in the bone marrow (Xiao et al., 2018).

In primary osteoblasts from a mouse model (*Pkd1* ^Oc-cKO^) in which *Pkd1* was specifically knocked out in skeletal tissues, a pronounced increase in lipogenic genes (*Pparg*, lipoprotein lipase, and adipocyte fatty acid-binding protein 2 [*aP2*]) and osteogenesis genes (*Runx2*, osteoprotegerin [*Opg*], and osteocalcin [OCN]), as detected by reverse transcription polymerase chain reaction (RT-PCR), was found. In addition, Oil Red O staining showed an increase in the number of adipocytes and fat droplets in decalcified bone sections, suggesting that BMSCs were more inclined to differentiate into adipocytes rather than maintain their original osteogenic function. This may lead to bone steatosis, which affects bone health and function (Xiao et al., 2010). The PC1-CTT can directly interact with the transcriptional co-regulator TAZ. Within the nucleus, TAZ functions as a transcriptional repressor of peroxisome proliferator-activated receptor γ (PPARγ) by binding to this key adipogenic regulator, thereby forming a PC1-CTT/TAZ/PPARγ ternary complex that suppresses the transcriptional activation of PPARγ target genes, such as *aP2* and *Lpl*. Chromatin immunoprecipitation (ChIP) assays have confirmed that PC1-CTT and TAZ are co-enriched at the PPARγ-binding site within the *aP2* promoter region, suggesting that this complex may mediate its inhibitory effect by blocking the transcriptional initiation activity of PPARγ (Xiao et al., 2018). However, current studies remain largely limited to the level of molecular interaction, and in-depth structural and signaling evidence regarding the mechanism of this complex is still lacking.

Qiu et al. revealed the important role of kinesin family member 3A (*Kif3a*) in regulating the imbalance between osteogenesis and lipogenesis induced by the deletion of *Pkd1* through a series of experiments. They found that the differentiation of BMSCs into adipocytes was markedly increased in *Pkd1* ^+/△^ heterozygous mice, whereas no significant change was observed in single *Kif3a* ^+/△^ heterozygous mice. However, when both mutations coexisted in the same mouse (*Pkd1* ^+/△^; *Kif3a* ^+/△^), the tendency of BMSCs to differentiate into adipocytes was considerably suppressed, indicating a reversal effect. This suggests that the *Kif3a* mutation may play a compensatory role in a *Pkd1*-deficient background, thereby alleviating skeletal diseases caused by *Pkd1* deletion by inhibiting the differentiation of BMSCs into adipocytes (Qiu et al., 2010). This finding provides a new avenue for treating related diseases.

### 3.2 Role of PC-1 in the regulation of osteoblast mineralization

Xiao et al. detected the expression of *Pkd1* in MC3T3-E1 osteoblasts and MLO-Y4 osteoblasts and confirmed the presence of its encoded product (PC-1) in osteoblasts and osteoclasts (Xiao et al., 2006). Further studies revealed that mice with a pure mutation in *Pkd1* showed spina bifida, osteoarticular dysplasia, and a pronounced delay in the processes of endochondral and intramembranous ossification at both embryonic and neonatal stages, suggesting that the deletion of *Pkd1* had a severe impact on skeletal development (Lu et al., 2001). In *Pkd1* gene-pure mutant mouse embryos, alizarin red/alcian blue staining showed reduced body size, decreased alkaline phosphatase (ALP) activity, and reduced mineralization of the ECM. This suggests that deletion of *Pkd1* leads to a weakening of the mineralization process or a delay in calcification, which results in considerable inhibition of skeletal growth that persists after birth (Xiao et al., 2006). Genes for osteoblast transcription factors (RUNX2-II, OCN, and OPG), osteoclast transcription factors (receptor activator of nuclear factor-kappa B ligand [RANKL]), and resistant acid phosphatase of tartrate (TRAP) were detected using RT-PCR. TRAP mRNA expression levels were markedly decreased (Xiao et al., 2006). These results suggest that the impaired bone formation process is not caused by enhanced bone resorption but by a notable reduction in the bone formation rate (Xiao et al., 2006).

To investigate the specific role of *Pkd1* in osteoblast differentiation, bone formation, bone maintenance, and its spatiotemporal specificity, *Pkd1* ^Oc-cKO^ and *Pkd1* ^Dmp1-cKO^ mouse models were constructed (Xiao et al., 2011; Xiao et al., 2010). Both models showed considerable weight loss and decreased bone density, indicating that deletion of *Pkd1* resulted in pronounced phenotypic changes, both at the early (*Dmp1* model) and late (Oc model) stages of osteoblast differentiation (Xiao et al., 2011; Xiao et al., 2010). Moreover, these findings suggest that *Pkd1* plays an important role in osteogenesis and is not limited to a specific stage. This further highlights the importance of *Pkd1* in bone biology and its potential as a therapeutic target.

To further validate the role of *Pkd1* in osteoblasts, a notable reduction in ALP activity, calcium deposition in the ECM, and the osteoblast differentiation marker RUNX2 was observed in the culture medium of *Pkd1* ^Dmp1-cKO^ and *Pkd1* ^Oc-cKO^ primary osteoblasts in an *in vitro* study (Xiao et al., 2011; Xiao et al., 2010). Additionally, RT-PCR showed decreased mRNA expression levels of RANKL, TRAP, and matrix metalloproteinase-9, further confirming that the main reason for the effect of *Pkd1* on bone loss was a decrease in the rate of bone formation rather than an increase in bone resorption (Xiao et al., 2011; Xiao et al., 2010). Notably, in the *Pkd1* ^m1Bei^ mutant mouse model, the double mutant showed a greater decrease in *Runx2* expression than that of the single mutant (Xiao et al., 2006). In *Pkd1* ^Oc-cKO^ knockout mice, there was a greater loss of trabecular and cortical bone in the femur, tibia, and lumbar spine than in *Pkd1* ^flox/m1Bei^ mice (Xiao et al., 2010). Mineral attachment rates in *Dmp1*-Cre, *Pkd1* ^flox/+^, and *Pkd1* ^Dmp1-cKO^ mice decrease with decreasing *Pkd1* gene dose (Xiao et al., 2011). The aforementioned studies not only revealed that *Pkd1* expression showed a significant gene dosage effect but also confirmed the critical role of *Pkd1* in maintaining skeletal homeostasis.

In exploring the specific mechanism of action of the PC-1 protein, researchers found that targeted deletion of *PKD1* in osteoblasts led to RUNX2-mediated osteoblast developmental abnormalities and bone reduction, suggesting that PC-1 can stimulate osteoblast maturation and proliferation through the activation of RUNX2 transcriptional activity (Xiao et al., 2023; Dalagiorgou et al., 2017). *RUNX2* includes two promoters, P1 and P2, which are responsible for the transcriptional activities of RUNX2-II and RUNX2-I, respectively (Xiao et al., 2003; Zhang et al., 2009). In *Runx2-II* ^−/−^ mice, deletion of *Runx2-II* pronouncedly affects skeletal growth and development, leading to symptoms such as growth retardation and dwarfism (Xiao et al., 2004). Serum levels of biochemical indicators, such as OCN, OPG, RANKL, and TRAP were suppressed. This suggests that the deletion of *Runx2-II* inhibited both bone formation and bone resorption processes, which led to abnormalities in bone metabolism, indicating the critical role of *Runx2-II* in bone homeostasis (Xiao et al., 2005).

In both embryonic and 6-week-old mouse skeletons, two-heterozygous *Pkd1* ^m1Bei^/*Runx2-II* ^+/−^ mice exhibited cumulative reductions in bone mass and BMD compared with that in single-heterozygous *Pkd1* ^m1Bei^ or *Runx2*-II^+/−^ mice. This further confirms the regulatory role of PC-1 on RUNX2 (Xiao et al., 2005). However, there was no significant change in the thickness of the cortical bone, which is the main expression and functional region of the P2-RUNX2-I subtype, indicating that PC-1 regulates the two RUNX2 types differently (Xiao et al., 2005). In mouse embryos with progressively decreasing doses of *Pkd1*, the mRNA expression levels of *Runx2-II* decreased pronouncedly with decreasing gene doses, whereas the mRNA expression of *Runx2-I* remained relatively stable (Xiao et al., 2006). In addition, co-transfection of PC1-AT (PC-1 C-terminal overexpression construct containing only the convoluted helix region) overexpression vector with P1 or P2 promoter-reporter gene vectors into MC3T3-E1 osteoblasts revealed that PC1-AT specifically activated the activity of the P1-*Runx2-II* promoter but did not affect the P2-*Runx2-I* promoter. This further demonstrated that PC-1 had clear specificity in its regulatory effect on *Runx2*, acting only on P1-*Runx2-II* and not P2-*Runx2-I* (Xiao et al., 2008).

To further investigate the mechanism by which the PC-1 protein activates signaling by RUNX2, Xiao et al. designed a series of PC-1 C-terminal overexpression constructs. These constructs included the following: PC1-LT comprising the entire C-tail of the IgG CH2-CH3 region, transmembrane region, G protein activation region, and the convoluted helix region required for coupling with PC-2; PC1-HT with a limited G protein signaling structural domain; PC1-LS containing only the G protein signaling structural domain; and PC1-AT (Xiao et al., 2006). Co-transfection of these PC-1 C-terminal constructs with *Runx2* P1 promoter-reporter genes into MC3T3-E1 osteoblasts and MLO-Y4 osteoblasts resulted in a remarkable increase in *Runx2* P1 promoter activity. This enhancement was pronounced in the PC1-HT and PC1-AT constructs, which contained convoluted structural domains (Xiao et al., 2006). This result suggests that the C-terminus of PC-1 can activate the transcriptional activity of *Runx2*, with the convoluted helical domain being crucial for the enhancement effect. The PC1-AT construct considerably enhanced the expression of endogenous *Runx2* in MC3T3-E1 osteoblasts and promoted the expression of bone formation-related genes, such as *Ocn* and *Opg* (Xiao et al., 2006).

The PC-1 protein plays an important role in regulating osteoblast differentiation and bone formation, specifically by affecting the activity of RUNX2. Thus, RUNX2 is a key molecule in the study of PC-1 and osteoblast function and bone formation mechanisms (Gargalionis et al., 2024).

### 3.3 Role of PC-1 in the regulation of osteoclast activity

Osteoclasts are multinucleated cells originating from hematopoietic stem cells, whose differentiation is mainly regulated by macrophage colony-stimulating factor and RANKL, which degrade bone tissue by releasing acids and proteolytic hydrolases on bone surfaces (Teitelbaum, 2000; Veis and O'Brien, 2023). Although existing studies on PC-1 in bone have mainly focused on osteoblasts (Xiao et al., 2023), some have shown that PC-1 can directly regulate osteoclastogenesis and bone resorption, thereby revealing a novel role for PC-1 in bone remodeling (Huang et al., 2024).

During osteoclast differentiation, *PKD1* mRNA expression levels gradually increase, showing active expression in osteoclasts (Huang et al., 2024). Therefore, we investigated its role in osteoclast differentiation and function. Huang et al. constructed three knockout mice models: *Pkd1* ^BMM△^ (early; macrophage stage), *Pkd1* ^TRAP△^ (middle to late; osteoclast differentiation process), and *Pkd1* ^CTSK△^ (late; mature osteoclast) (Huang et al., 2024). These models helped in understanding the complex role of *Pkd1* in different stages of osteoclast development and function. Although *Pkd1* mRNA levels were markedly reduced in these models, bone trabeculae, bone volume ratios, and cortical bone thickness were markedly increased compared with those in the controls (Huang et al., 2024). However, the number of osteoclasts on the surface of the bone trabeculae and serum levels of type I collagen carboxy-terminal peptide (Type I collagen C-telopeptide, CTX-1) was markedly reduced, but the number of osteoblasts on the surface of the bone trabeculae did not change significantly (Huang et al., 2024). This suggests that *Pkd1* knockdown, specifically in osteoclasts, results in diminished bone resorption activity and unaffected bone formation, a change that may be beneficial in preventing osteoporosis or improving bone strength. Furthermore, bone marrow macrophages (BMM) isolated from *Pkd1* ^TRAP△^ mice exhibited remarkable inhibition of bone resorption by deletion of *Pkd1* (Huang et al., 2024). This was evidenced by a reduction in the number of TRAP-positive multinucleated cells, impaired F-actin ring formation, and markedly reduced resorption activity *in vitro* bone sections (Huang et al., 2024). Both *in vitro* and *in vivo* studies have confirmed the critical role of *Pkd1* in regulating bone resorption.

The specific mechanism of osteoclast activation by the PC-1 protein is mainly regulated by its CTT fragment (Huang et al., 2024). Ectopic expression of PC1-CTT in BMMs results in enhanced expression of cathepsin K (CTSK), nuclear factor of activated T-cells cytoplasmic 1 (NFATC1), and receptor activator for nuclear factor-κB (RANK) genes, and an increase in the number of TRAP-positive osteoclasts (Huang et al., 2024). In contrast, when BMMs were treated with DAPT, a compound that inhibits prosecretory enzymes and restricts the cleavage and release of PC-1 CTT, the expression of osteoclast-related genes was reduced, and the formation of TRAP-positive osteoclasts was inhibited (Huang et al., 2024). This suggests that the PC-1 CTT controls osteoclast activity through cleavage and release. In addition, transcriptome RNA sequencing (RNA-seq) analysis of osteoclasts transfected with *PKD1* small interfering RNA (siRNA) and pathway enrichment analysis of the Kyoto Encyclopedia of Genes and Genomes database showed that signaling pathways associated with osteoclasts were inhibited in samples with downregulated *PKD1* expression (Huang et al., 2024). However, the specific mechanism of action requires further study.

## 4 Role of PC-1 in mediating mechanotransduction in bone

### 4.1 PC-1 mediates the process of bone mechanical force regulation

In the skeletal system, mechanotransduction is a central mechanism that regulates bone tissue homeostasis and adaptive remodeling. Bone tissue is a highly dynamic structure that is continuously regulated by mechanical stress impacting its morphology and function. Osteoblasts sense physical stimuli, such as stretching and pressure, from the ECM or the surrounding microenvironment and translate them into intracellular biochemical signals (Nims et al., 2022). Notably, the transmembrane protein complex, PC-1, has numerous extracellular domains that bind to ECM components, sense extracellular mechanical signals, and transmit these signals to the cell, thereby regulating the activity of downstream signaling molecules and mediating physiological responses to mechanical stimuli (Qian et al., 2005; Nigro and Boletta, 2021). Therefore, PC-1 is considered one of the core molecules that integrates mechanical force signals (Xiao et al., 2011).

A study by Xiao et al. found that the deletion of *Pkd1* in mature osteoblasts leads to impaired mechanosensing *in vivo* (Xiao et al., 2011). Ulnar strain gauge testing of 16-week-old *Pkd1* ^Dmp1-cKO^ mice revealed that the peak compressive strain on the lateral side of the ulnar midshaft was linearly related to the peak tension strain in the *Pkd1* ^Dmp1-cKO^ and *Pkd1* ^*flox*/+^ groups; however, the strain values were higher in the *Pkd1* ^Dmp1-cKO^ group (Xiao et al., 2011). These findings suggest that deleting *Pkd1* leads to a reduction in skeletal stiffness, making the skeleton more susceptible to deformation under external forces. In addition, the femoral three-point bending test showed that, although the maximum stress increased, there was no notable difference in the bending stiffness, failure energy, or maximum force. These results suggest that in the case of damage to mechanotransduction caused by *Pkd1* deletion, the bone may maintain its overall mechanical properties by adjusting its geometry and/or microstructure (Xiao et al., 2011). Such adaptive changes may help compensate for the potential negative effects of gene deletion.

Shalish et al. revealed the critical role of PC-1 in mechanotransduction. Orthodontic tooth movement experiments in mice revealed that the molar teeth of wild-type mice exhibited the expected movement after the application of orthodontic forces, whereas the molar teeth of mutant mice lacking PC-1 did not move (Shalish et al., 2014). Further studies showed that in wild-type mice, active osteoclasts were observed on the compressed side after the application of force, but in PC-1 mutant mice, the osteoclasts did not appear on the expected bone surfaces or in the compressed area but were distributed in the bone marrow. This suggests that PC-1 may act as a mechanosensor and regulate osteoclast formation and activity (Shalish et al., 2014). In addition, PC-1 knockout showed noteworthy effects in response to bone loss under weightlessness or simulated weightlessness (Huang et al., 2024). Specifically, wild-type mice with unweight-bearing distal femurs showed a marked reduction in bone mass and an increase in the number of osteoclasts on the bone surface in the hanging test, but similar changes were not observed in a model with a specific knockdown of *Pkd1* in BMMs (*Pkd1* ^BMM△^) and TRAP-positive osteoclasts (*Pkd1* ^TRAP△^) (Huang et al., 2024). These findings further demonstrate that the PC-1 protein plays a key role in osteoclasts in response to mechanical loads (Huang et al., 2024).

Taken together, these studies not only reveal the central role of PC-1 in regulating osteoblast function but also indicate its potential mechanisms in adapting to mechanical stress and maintaining bone mechanical properties.

### 4.2 Role of PC-1 in mediating bone mechanical forces for bone formation

Osteoblasts are a key cell type in bone formation that sense and respond to external mechanical force signals to initiate the synthesis and mineralization of bone matrix (Wang et al., 2020). The effects of mechanical forces on bone formation are bidirectional. Appropriate mechanical stimulation promotes bone formation and increases bone mass and bone density, whereas excessive mechanical loading may lead to bone damage and fracture, thereby inhibiting bone formation (Ma et al., 2023; Sun et al., 2021). In this process, PC-1 plays a central role as a mechanical force-sensing protein (Wang et al., 2014; Xiao et al., 2011).

*In vivo* studies by Xiao et al. using RT-PCR found that the expression of *Runx2-II*, cyclooxygenase-2 (*Cox2*), cellular Jun (*c-Jun*), Wnt family member 10B (*Wnt10b*), frizzled class receptor 2, and Axis inhibition protein 2 (*Axin2*) were markedly increased in wild-type mice (Xiao et al., 2011). However, the *Pkd1* ^Dmp1-cKO^ group showed no response (Xiao et al., 2011). This suggests that deleting *Pkd1* impaired the sensitivity and response of bone tissue to mechanical stimuli. In addition, the measurement of the periosteal mineralization rate further revealed the effect of the *Pkd1* dose on bone formation; the lower the *Pkd1* gene dose, the lower the mineral deposition rate and the weaker the response to mechanical stress, which was positively correlated with the *Pkd1* transcript level (Xiao et al., 2011).

Under fluid shear stimulation, osteoblasts in *Pkd1*-deficient osteoblasts showed notable attenuation of intracellular calcium ion responses (Xiao et al., 2011). Moreover, both mRNA and protein levels of PC-1 were considerably decreased in *Pkd1* small hairpin RNA (shRNA)-disrupted cells. After 1 h of mechanical tensile stress, the gene and protein expression levels of PC-1, RUNX2, osteoblast-specific transcription factor (osterix), OCN, and osteopontin were reduced, as was the intracellular Ca+ ion concentration (Wang et al., 2014). Further evidence is required to determine the important role of PC-1 in mechanosignaling.

The results of both *in vitro* and *in vivo* studies indicate that PC-1 plays a key role in bone mineralization and mechanosensing, considerably affecting the ability of bone tissue to sense mechanical signals and its response activity. Future studies should further reveal the molecular mechanism of PC-1 in bone tissue mineralization and mechanosensing. Moreover, they should explore how to optimize the bone formation process by regulating the PC-1 signaling pathway.

### 4.3 Role of PC-1 in mediating bone mechanical force regulation of bone resorption

The conventional view is that osteoclasts do not respond directly to mechanical forces; however, they indirectly regulate bone resorption through paracrine signals from other cells, such as osteoblasts and osteocytes. However, osteoclasts can sense mechanical forces directly. *In vitro* studies, BMMs directly affected osteoclast activation in the presence of fluid shear stress (FSS) (Bratengeier et al., 2020). *In vivo* studies, mechanical stimulation controlled osteoclast function by regulating Ca^2+^-activated chloride (Cl^−^) channel anoctamin 1; however, the exact mechanism was not clear (Sun et al., 2023). Moreover, PC-1-mediated mechanical forces directly regulate osteoclast activity (Huang et al., 2024).

*In vitro* studies of suspended mice showed an increase in the number of TRAP-positive osteoclasts in BMMs and an upregulation of the expression of osteoclast-specific genes *Ctsk*, *Nfatc1*, and *Rank* (Huang et al., 2024). Notably, under conditions of mechanical unloading, *Pkd1* expression increases, but the expression of *Piezo1* decreases significantly, which provides new perspectives and ideas for a comprehensive understanding of the mechanisms of skeletal mechanoreception (Huang et al., 2024). In addition, BMMs were cultured under static or simulated microgravity conditions, and quantitative PCR analysis revealed that the mRNA expression levels of *Ctsk* and *Nfatc1* genes were notably higher in microgravity conditions than in static controls. These results suggest that mechanical unloading induces osteoclastogenesis (Huang et al., 2024). Under the effect of FSS, the expression of *Ctsk*, *Nfatc1*, and *Rank*, which are closely related to the function of osteoclasts, showed a notable decrease in the control group. However, no significant changes in the expression of these genes were observed in osteoblasts, specifically when knocking down *Pkd1*, despite being subjected to the same FSS effect (Huang et al., 2024). This suggests that *Pkd1* plays a critical regulatory role for FSS in osteoblasts. *Pkd1* expression rises under mechanical unloading and microgravity and promotes bone resorption, whereas it is inhibited in the presence of FSS.

Although the aforementioned study suggests that PC-1 regulates osteoclast activity through mechanical forces, its specific molecular mechanism remains to be explored in depth. To further reveal the central role of PC-1 in bone resorption, future studies should focus on the mechanism by which PC-1 integrates mechanical force signaling and regulates osteoclast differentiation and activity through other ion channels.

## 5 Mechanisms of PC-1 regulation of bone reconstruction

### 5.1 PC-1-mediates CaN/NFAT signaling pathway

CaN (also known as calmodulin), a heterodimeric protein comprising two subunits, is an important protein in intracellular calcium signaling responses and can be activated by calcium ions (Ulengin-Talkish and Cyert, 2023). The NFAT family is a substrate for CaN, and members of the NFAT family migrate to the nucleus after dephosphorylation catalyzed by CaN (Jain et al., 1993). The PC-1 C-tail activates the CaN/NFAT signaling pathway (Puri et al., 2004; Dalagiorgou et al., 2013).

Activation of the CaN/NFAT signaling pathway in HEK293T cells by PC-1 requires both an intact G protein binding and activation region and the release of the intracellular Ca^2+^ reservoir coupled with the influx of extracellular Ca^2+^ (Puri et al., 2004). This process leads to a notable increase in the cytoplasmic Ca^2+^ concentration, which is a key step in CaN activation (Puri et al., 2004).

The activated CaN catalyzes the dephosphorylation of NFAT and its translocation into the nucleus, thereby initiating the transcription of specific genes (Puri et al., 2004). When using Ca^2+^, the inhibitor notably inhibited PC-1 C-tail-mediated NFAT activation, whereas using CsA (an inhibitor of CaN) blocked PC-1 C-tail-induced NFAT activation, demonstrating that NFAT activation strictly depends on the catalytic action of CaN (Puri et al., 2004). Notably, PC-2 does not play a role in this process (Puri et al., 2004). PC-1 plays a similar role in the human model. Mechanical stretching of human periodontal fibroblasts (hPDL) *in vitro* revealed that PC-1 expression increased under stretch, whereas pNFATc1 showed the opposite change; treatment of mechanically stretched cells with CsA (CaN/NFAT pathway inhibitor) and anti-Ig-PKD antibody (PC-1 inhibitor) reversed mechanical stretch-induced changes in NFAT and phosphorylated NFAT levels (Dalagiorgou et al., 2013).

**FIGURE 2:**
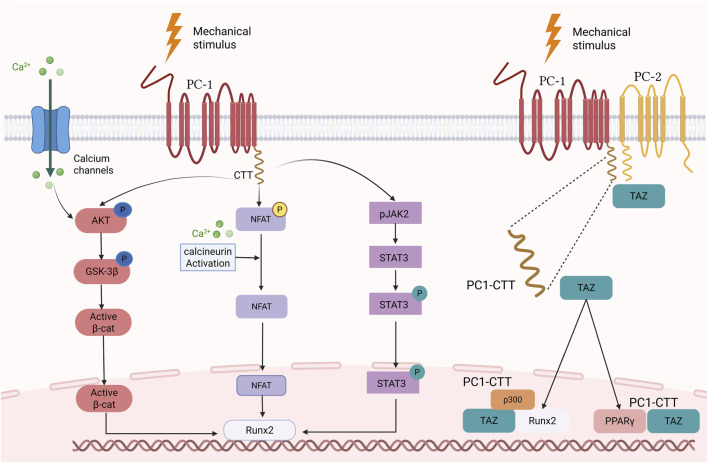
PC-1 signal pathway mediated by mechanical force. PC-1-CTT, Polycystin-1 C-Terminal Tail; GSK-3β, Glycogen synthase kinase-3 beta; JAK2, Janus kinase 2; pJAK2, Phosphorylated Janus kinase 2; NFAT, Nuclear factor of activated T cells; PC-1, Polycystin-1; PC-2, Polycystin-2; STAT3, Signal transducer and activator of transcription 3; TAZ, Transcriptional coactivator with PDZ-binding motif; Active β-cat, Active β-catenin; p300, p300 transcriptional coactivator; PPARγ, Peroxisome Proliferator-Activated Receptor Gamma.

Furthermore, mechanical stretching not only activated the PC-1-mediated CaN/NFAT signaling pathway but also markedly increased RUNX2 mRNA expression (Dalagiorgou et al., 2013) (Figure 2). The RUNX2 mRNA expression level was low in unstretched hPDLs and in stretched hPDLs treated with PC-1 inhibitory antibody; after stretching of the hPDL, the expression level of RUNX2 elevated by more than twofold (Dalagiorgou et al., 2013). Interestingly, the expression levels of NFATc1 and RUNX2 did not increase immediately but only after 0.5 h. This time delay may reflect the complexity of signaling and gene expression regulation. Studies on the effects of PC-1 on the CaN/NFAT signaling pathway in osteoblasts and osteoclasts are rare, and the mechanism of action remains to be further elucidated.

### 5.2 PC-1 mediates the JAK/STAT signaling pathway

The JAK/STAT pathway is a key mechanism of intracellular signaling and plays an important role in bone remodeling and osteoblast differentiation (Zhou et al., 2011; Li, 2013). In primary human osteoblasts, the PC-1 C-terminal tail (CTT) regulates osteoblast responses to mechanical stress by forming a complex with JAK2 and phosphorylated JAK2 (pJAK2) (Dalagiorgou et al., 2017). Specifically, in a static environment, PC-1 CTT forms a complex with JAK2; however, when mechanical stretch is applied, PC-1 binding to JAK2 is considerably attenuated, whereas PC-1 CTT complexing with pJAK2 is markedly increased, suggesting that mechanical stress specifically enhances the interaction of PC-1 with pJAK2 (Dalagiorgou et al., 2017). In an *in vitro* study of primary human osteoblasts, mechanical stretch resulted in a considerable increase in the level of pJAK2, as shown by Western blot (WB) analysis, and was accompanied by enhanced phosphorylation and intranuclear translocation of STAT3. This suggests that PC-1 further regulates the function of STAT3 through the activation of JAK2 (Dalagiorgou et al., 2017).

STAT3 is a key transcription factor in the JAK2 signaling pathway (Xiao et al., 2022). Stimulated by mechanical stretching, pJAK2 activates STAT3, thereby promoting its phosphorylation and intranuclear translocation (Dalagiorgou et al., 2017). Chromatin immunoprecipitation (ChIP) experiments further showed that mechanical stress promoted the entry of phosphorylated STAT3 into the nucleus, where it binds to the promoter region of *RUNX2* and activates the expression of RUNX2 (Dalagiorgou et al., 2017). *RUNX2* is an important transcription factor involved in osteoblast differentiation and bone formation. PC-1 ultimately promotes RUNX2-mediated osteoblast gene expression by regulating the JAK2/STAT3 signaling pathway. Notably, after pretreatment of osteoblasts with the JAK2 inhibitor AG490 or the PC-1 inhibitor anti-IgPkd1, mechanical stretch-induced elevation of pJAK2 levels was suppressed, suggesting that JAK2 activation depends on PC-1 (Dalagiorgou et al., 2017). JAK2 and STAT3 inhibitors also block the intranuclear translocation of phosphorylated STAT3, further demonstrating the important role of PC-1 in the JAK/STAT signaling pathway (Dalagiorgou et al., 2017).

PC-1 CTT binds specifically to JAK2, a process that activates JAK2 and leads to the phosphorylation of STAT3, which subsequently translocates to the nucleus and binds to the promoter region of *RUNX2*, thereby affecting osteoblast differentiation and bone formation (Dalagiorgou et al., 2017) (Figure 2). Thus, the PC1-JAK2/STAT3 signaling pathway plays an important role in bone biology and may be a potential therapeutic target for interventions in skeletal diseases. Future studies should explore how to optimize the therapeutic potential of this pathway by regulating the interaction between PC-1 and JAK2.

### 5.3 PC-1-mediated AKT/β-catenin signaling pathway

Mechanical stimulation has been shown to regulate osteoblast differentiation and maturation through multiple signaling pathways, in which the influx of extracellular Ca^2+^ and regulation of intracellular Ca^2+^ play a key role (Qin et al., 2020). Notably, PC-1 mediates this process (Puri et al., 2004). Intracellular Ca^2+^ regulates AKT activity (Porto Ribeiro et al., 2022), and AKT inhibits the activity of glycogen synthase kinase (GSK)-3β by phosphorylation (Zhong et al., 2023). GSK-3β is a kinase that promotes the degradation of β-catenin, which inhibits the activity of the Wnt/β-catenin signaling pathway (Zhong et al., 2023). Based on these findings, Wang et al. proposed a new mechanism by which PC-1 regulates the osteoblast response to mechanical forces by mediating interactions between Ca^2+^, AKT, GSK-3β, and β-catenin (Wang et al., 2014). Therefore, investigating the specific operation of PC-1 in the AKT/β-catenin signaling pathway is important.

In an *ex vivo* assay, after mechanical stress was applied to the mouse osteoblast cell line MC3T3-E1, WB analysis revealed that the basal phosphorylation levels of AKT and GSK-3β were markedly elevated, suggesting that mechanical stress effectively activated the AKT signaling pathway, which promoted the phosphorylation of GSK-3β. Immunofluorescence staining further confirmed that mechanical stress considerably upregulated β-catenin expression and triggered the accumulation of active β-catenin in the nucleus, which ultimately led to an increase in the expression of *Runx2*, a key step in osteoblast differentiation and maturation (Wang et al., 2014). However, when the AKT-specific inhibitor AKTi-1/2 was used, the mechanical stress-induced phosphorylation levels of AKT and GSK-3β were notably inhibited, and immunofluorescence results showed that mechanical stress could no longer induce the accumulation of active β-catenin in the nuclei of cells (Wang et al., 2014). These results suggest that mechanical stress promotes osteoblast differentiation and maturation through the AKT/GSK-3–β/β-catenin signaling pathway.

This series of positive signaling processes was considerably inhibited in PC-1 loss-of-function osteoblasts. In primary osteoblasts from *Pkd1* ^Oc-cko^ knockout mice, phosphorylation of AKT and GSK-3β in the basal state was markedly lower than that in the normal group, and the expression of β-catenin was at a lower level, as shown by key components of the Wnt signaling pathway; WNT10B, AXIN2, COX2, and RUNX2-II expression levels were decreased, indicating the importance of PC-1 protein in regulating these signaling pathways (Xiao et al., 2010). *In vitro* studies, inward calcium flow in *Pkd1*-shRNA cells was pronouncedly restricted under mechanical stress. Although mechanical stress notably increased the phosphorylation levels of AKT in control cells, the phosphorylation levels of AKT were markedly reduced in the PC-1-deficient group (Wang et al., 2014). In addition, PC-1 deficiency resulted in decreased GSK-3β phosphorylation levels and reduced accumulation of β-catenin in the nucleus, which inhibited the expression of osteogenic genes, such as *Runx2* (Wang et al., 2014). Notably, when the calcium ion carrier A23187 was applied to *Pkd1* shRNA cells, the phosphorylation of AKT and GSK-3β as well as the expression of active β-catenin in the nucleus were remarkably upregulated, implying a critical role of calcium ion signaling in maintaining the positive AKT/GSK-3 β/β-catenin transduction (Wang et al., 2014).

The critical role of PC-1 in the AKT/β-catenin signaling pathway provides a new direction for the treatment of skeletal diseases. Therefore, future studies could explore the restoration of the normal differentiation function of osteoblasts by targeting and regulating the AKT/β-catenin pathway. This will aid in developing potential therapeutic options for skeletal diseases, such as osteoporosis.

### 5.4 Mechanical force-driven PC-1/TAZ composite bone reconstruction

The Hippo signaling pathway plays a key role in the regulation of cell proliferation and organ size, considerably impacting osteoblasts, osteoclasts, and osteogenic differentiation of mesenchymal stem cells (Wang et al., 2020; Li et al., 2018; Lorthongpanich et al., 2019). TAZ protein (encoded by *WWTR1*) is a critical downstream effector of the Hippo pathway and acts as a transcriptional co-activator (Wang et al., 2023). The interaction of PC-1 with TAZ provides a new perspective on the regulation of bone development and adipogenesis and reveals a novel mechanosensing mechanism during bone formation in mice (Xiao et al., 2023; Merrick et al., 2019; Xiao et al., 2018).

#### 5.4.1 PC-1/TAZ mediates mechanical force regulation of bone formation mechanisms

Reduced jaw mineralization and tail curvature were observed in a *Pkd1* knockdown zebrafish model (Merrick et al., 2019). These undesirable phenotypes were successfully reversed by injecting the knockdown zebrafish with mRNA encoding PC1-CTT or constitutively active TAZ (S89A). However, in the absence of TAZ, PC1-CTT expression alone did not rescue these phenotypes (Merrick et al., 2019). Interestingly, TAZ independently restored bone density and corrected caudal curvature in the context of impaired *Pkd1* expression. These experimental results illustrate the critical regulatory role of PC-1 in skeletal development and morphological maintenance and suggest that PC-1 may act as an upstream regulator of TAZ (Merrick et al., 2019).

Using co-immunoprecipitation (Co-IP) assays, Xiao et al. identified that complexes between TAZ and PC-1 and PC-2 could be formed in HEK-293T cells (Xiao et al., 2018). Subsequently, Merrick et al. investigated the mechanism of interaction between PC-1 and TAZ in HEK293 cells. They focused on the last 91 amino acids of the PC-1 CTT (p91 region), which contain a convoluted helical structural domain. Through GST pull-down experiments, p91 was found to form a complex with TAZ, suggesting that this region is a key site for PC-1 interaction with TAZ. This finding provides important clues for understanding the molecular mechanism by which PC-1 regulates the function of TAZ (Merrick et al., 2019).

*Pkd1* and *Taz* co-deficiency led to a superimposed effect on reduced BMD (Xiao et al., 2023; Xiao et al., 2018) in double heterozygous *Taz* ^+/−^; *Pkd1* ^+/−^ mice and double-knockout *Pkd1*/*Wwtr1* ^Oc-cKO^ mice. Micro-CT analysis showed that bone volume and trabecular and cortical bone loss were more pronounced in double-knockout mice than in single-knockout mice. RT-PCR analysis showed that the osteoblast gene profiles of *Runx2-II*, *Opg*, dentin matrix protein 1 (*Dmp1*), and other osteogenic genes were markedly decreased in double-knockout mice (Xiao et al., 2023; Xiao et al., 2018). However, common deletions had different effects on adipocytogenesis. A considerable increase in the number of adipocytes and the volume of fat droplets was observed in the decalcified femurs and tibiae of *Taz* ^+/−^; *Pkd1* ^+/−^ double-knockout mice stained with oil red and OsO4 staining (Xiao et al., 2018). However, there was no significant cumulative effect of PPARγ, aP2, and lysophosphatidylinositol in double-knockout *Pkd1*/*Wwtr1* ^Oc-cKO^ mice compared with that in single-knockout mice (Xiao et al., 2023). This suggests a complex mechanism of interaction between PKD1 and TAZ in regulating the expression of these adipogenesis-related genes; however, the mechanism of action is unclear.

PC1-CTT interacts with TAZ to co-regulate the activity of two key transcription factors: first, it promotes osteoblast differentiation by affecting RUNX2 regulation of the promoter of an osteoblast-specific gene (OCN); and second, it represses adipocyte differentiation by affecting PPARγ regulation of the promoter of an adipocyte-specific gene (*aP2*), which affects the differentiation process of stem cells (Xiao et al., 2018). In C3H10T1/2 mesenchymal stem cells (MSC), immunoprecipitation and ChIP assays showed that TAZ and PC1-CTT not only bind to RUNX2 at the OSE2 site in the promoter region of OCN to enhance the expression of osteogenic genes but also bind to PPARγ at the promoter region of *aP2* (the target gene of PPARγ) at the ARE6 site to inhibit its expression, thereby reducing adipocyte differentiation (Xiao et al., 2018). These *in vitro* and *in vivo* studies demonstrated that the interaction of PC1-CTT with TAZ affects osteoblast and adipocyte differentiation by co-regulating OCN and *aP2* promoter activity (Figure 2).

PC-1 and TAZ can be used as novel mechanical sensors (Xiao et al., 2023). In a mechanical tibial loading study of *Pkd1*/*Wwtr1* ^Oc-cko^ mice, tibial mechanotransduction factors, osteogenic factors, and lipogenic factors were found to not be significantly altered in mice with *Pkd1* and *Wwtr1* knockout, nor was there a significant change in calcium xanthophyll double-labeling of the mineralization rate compared to wild-type pairs of mice, suggesting that in the absence of *Pkd1* and *Wwtr1* in osteoblasts, the response of bone tissue to mechanical stimuli may be considerably affected. This suggests that PC-1 and TAZ may play important roles in the mechanotransduction process in bone (Xiao et al., 2023). Future studies are needed to explore further details of this pathway to provide new ideas for the treatment of bone-related diseases and disorders of fat metabolism.

Although the aforementioned study revealed the critical role of PC-1/TAZ in bone formation, there are still numerous questions. The molecular mechanisms by which PC-1/TAZ synergistically sense and conduct mechanical forces to regulate osteogenesis and adipogenesis need to be further explored. In addition, the specific functional differences between PKD1 and TAZ in different cell types, as well as their different responses to mechanical force sensitivity, are important directions for future research. An in-depth study of this pathway can provide new ideas for treating bone-related diseases and metabolic disorders.

#### 5.4.2 Regulation of osteoclast activity by the PC-1/TAZ complex

The interaction between PC-1 and TAZ promotes osteoclast differentiation. WB results showed that the knockdown of *Pkd1* in osteoclasts resulted in a decrease in TAZ in the nucleus. This indicates inhibition of nuclear translocation, whereas overexpression of the CTT of PC-1 facilitated the translocation of TAZ to the nucleus. Co-IP assays revealed that both PC-1 and its CTT could form a complex with TAZ protein, which activates the transcription of osteoclast-related genes (Huang et al., 2024). ChIP-seq and ChIP-PCR analyses showed that TAZ bound to the promoter regions of the osteoclast-related genes *Siglec15*, *Ocstamp*, *Oscar*, and *Acp5*, directly regulating their transcription. Further analysis showed that TAZ is also bound to the *Pkd1* promoter and that silencing TAZ downregulated *Pkd1* mRNA expression. These findings reveal a positive feedback mechanism: activation of the PC1-TAZ axis promotes the expression of osteoclast-related genes and enhances the transcription of *Pkd1*, which plays a key role in regulating osteoclastogenesis and function (Huang et al., 2024).

Pharmacological intervention of the PC1-TAZ axis shows therapeutic potential (Huang et al., 2024). Zinc01442821, a specific small-molecule compound, inhibits the formation of PC1/PC2/TAZ complexes in MSCs, which reduces the expression of osteoclast-related genes and inhibits the formation of TRAP-positive multinucleated cells (Huang et al., 2024). In an ovariectomy-induced bone loss model, mice treated with Zinc01442821 showed a 30% increase in trabecular volume, 25% increase in trabecular thickness, and 45% decrease in the density of TRAP-positive cells on the surface of the bone compared with that in the control group (Huang et al., 2024). Notably, *Pkd1* knockdown in peripheral blood mononuclear cells (PBMC) from healthy volunteers inhibited the differentiation and formation of human osteoclasts. Zinc01442821 inhibits osteoclast differentiation in human PBMCs; hence, these results suggest that Zinc01442821 has great potential for the treatment of osteoclast-related osteoporosis (Huang et al., 2024).

#### 5.4.3 The mechanism by which PC-1/TAZ regulates osteochondral stem/progenitor cell differentiation

Periosteal stem/progenitor cells (PSPC) are a group of pluripotent stem cells in the inner layer of the periosteum that play key roles in bone growth, repair, and remodeling processes (Jeffery et al., 2022). These cells are capable of self-renewal and multidirectional differentiation into various cell types, such as osteoblasts, chondrocytes, and adipocytes (Jeffery et al., 2022; Duchamp de Lageneste et al., 2018). TAZ exhibits great potential for regulating the osteogenic and chondrogenic differentiation of PSPCs. By transfecting *Taz* siRNA into PSPC cells, the expression of *Taz* was markedly reduced, and bone gamma-carboxyglutamic acid protein (Bglap), aggregated proteoglycan (*Acan*), and II Collagen Type II Alpha 1 (*Col2a-1*) chain genes were expressed, and the differentiation capacity of PSPC cells was diminished; in a suspension mouse model, mechanical unloading pronouncedly inhibited nuclear translocation of TAZ (Liu et al., 2024). Similarly, in the *Pkd1*-*Ctsk*-CKO mouse model with deletion of *Pkd1*, a reduction in TAZ nuclear translocation was observed (Liu et al., 2024).

Compared with that in the control group, the overexpression of PC1-CTT notably enhanced the osteogenic and chondrogenic differentiation capabilities of PSPCs. This effect was reflected by the pronounced upregulation in the expression levels of osteogenesis-related genes (*ALP*, *RUNX2*, *Sp7* [Sp7 transcription factor], and *BGLAP*), as well as the chondrogenesis-related gene *ACAN* (Liu et al., 2024). In addition, when PSPCs were treated with DAPT, the expression of bone- and cartilage-associated genes was reduced, and the differentiation ability of chondrocytes was notably inhibited. These findings strongly suggest that inhibition of PC1-CTT release markedly reduces the osteogenic and chondrogenic differentiation potential of PSPCs (Liu et al., 2024).

Further studies found that Zinc01442821 could also modulate the repair of fractures through the PC1-TAZ axis (Liu et al., 2024). Micro-CT analysis revealed that Zinc01442821 increased BMD at the fracture site and improved the formation of mineralized cartilage scabs. In an *in vitro* study, Alcian Blue staining showed that treatment increased osteogenic differentiation as well as cartilage matrix formation in PSPCs and enhanced nuclear translocation of TAZ (Liu et al., 2024). However, *Pkd1* knockdown attenuated the positive effect of Zinc01442821 on the osteogenic differentiation of PSPCs, probably because the lack of *Pkd1* inactivated the stimulatory effect of Zinc01442821 on intracellular calcium and TAZ activation, resulting in attenuated osteogenic differentiation (Liu et al., 2024).

Taken together, these studies revealed the importance of the PC-1/TAZ axis in the periosteal stem/progenitor cell differentiation and fracture healing. However, the specific molecular mechanisms by which the PC-1/TAZ axis senses and integrates mechanical stress are not yet fully understood. Future studies should explore the signaling pathways through which PC-1 regulates osteogenic and chondrogenic differentiation *via* TAZ, especially its interactions with other signaling pathways. In addition, Zinc01442821 exhibits great potential as a therapeutic agent in fracture healing, and further optimization of its ability to modulate the PC-1/TAZ axis will contribute to the development of novel fracture treatment strategies.

## 6 PC-1 and bone-related diseases

### 6.1 Role of PC-1 in fracture healing

A fracture occurs when the integrity of the bone is compromised by external forces or pathological factors, and the healing process is critical for restoring the structure and function of the bone (Chandran et al., 2024). PSPCs play an indispensable role in fracture healing and are involved in bone regeneration and repair through both intramembranous and endochondral osteogenesis (Debnath et al., 2018; Tsukasaki et al., 2022). These cells participate in bone regeneration and repair, and mechanical loading accelerates healing in this process (Jeffery et al., 2022). CTSK is an important marker for recognizing PSPCs, and CTSK-positive cell populations include periosteal stem cells and progenitor cells, which play key roles in maintaining homeostasis and regenerating cortical bone (Zou et al., 2022; Debnath et al., 2018).

Periosteal stem/progenitor cells that organize proteinase K (CTSK^+^ PSPC) sense mechanical forces through PC-1 and regulate osteochondral formation and bone healing processes (Liu et al., 2024). CTSK-positive cell-specific knockout *Pkd1* (*Pkd1* ^Ctsk-CKO^) mice were obtained by crossing CTSK-CRE with *Pkd1* ^flox/flox^ mice. These mice exhibited pronounced thinning of the femoral cortex, a reduction in cortical bone volume, and a decrease in the number of OCN-positive osteoclasts on the cortical bone surface. However, in mice with a specific *Pkd1* knockout in osteoclasts (*Pkd1* ^Trap-CKO^), cortical bone thickness was markedly increased. This comparative result excludes the effect of functional changes in osteoclasts on the bone phenotype, thus clarifying the critical role of PKD1 in CTSK^+^ PSPCs in cortical bone formation and the maintenance of bone homeostasis (Liu et al., 2024). In an *in vitro* study, *Pkd1* siRNA was transfected into PSPCs, and a decrease in the expression levels of the osteogenesis-related genes *Runx2*, *Alp*, and *Bglap*, as well as the chondrogenesis-related gene *Acan*, was observed in PSPCs (Liu et al., 2024). These results further confirmed the irreplaceability of PKD1 in the osteochondral differentiation of PSPCs and its central role in cortical bone formation (Liu et al., 2024).

In the *Pkd1* ^Ctsk-CKO^ mouse model of mid-femoral transection fracture, micro-CT results showed that deletion of the *Pkd1* gene led to a reduction in the size of the healing tissue and a reduction in the ossification process. Safranin O/Fast green staining also showed a reduction in the size of the healing tissue and an impaired bone formation process (Liu et al., 2024). These results suggest that *Pkd1* deletion in PSPCs adversely affects the fracture healing process (Liu et al., 2024). In addition, in the suspension model, *Pkd* ^-Ctsk-CKO^ mice did not show a more severe delay in fracture healing compared with that of wild-type mice, which may be because the suspension model inherently reduces mechanical stimulation, and the *Pkd1* deletion did not further exacerbate the effect of mechanical unloading on fracture healing (Liu et al., 2024).

PC-1 regulates osteochondral differentiation through mechanotransduction in PSPCs and is an indispensable molecule in fracture healing. The role of PC-1 in sensing mechanical forces provides new perspectives for understanding fracture healing, as well as potential targets to treat fractures. Future studies should focus on the specific signaling mechanisms of PC-1 in PSPCs, especially the mechanism by which PC-1 regulates the molecular processes of bone and cartilage formation *via* the integration of external mechanical signals with intracellular signaling pathways.

### 6.2 Role of PC-1 in premature closure of human cranial sutures

Premature closure of cranial sutures is a serious cranial developmental abnormality that leads to craniosynostosis and elevated intracranial pressures (Wu and Gu, 2019). This pathology not only restricts the space for brain growth and increases intracranial pressure but may also affect the cognitive, motor, and other brain functions of the patient (Blum et al., 2022; Oussoren et al., 2018). The pathogenesis of premature closure of cranial sutures is complex and involves a variety of genetic and environmental factors, among which abnormal bone formation is considered an important causal factor (Mefford et al., 2010; Varvagiannis et al., 2013). PC-1 may be a potential target to treat premature closure of the cranial suture by regulating mechanical stress perception and cellular signaling in the bone (Kolpakova-Hart et al., 2008).

In animal model studies, deletion of *Pkd1* was found to significantly affect the development of the skull base cartilage. In *Wnt1*-Cre; *Pkd1* (*Pkd1* is knocked out in *Wnt1*-expressing cells of neural crest origin) mice, only the anterior skull base cartilage joint (PSS) showed premature closure, whereas the pterygoid-occipital cartilage joint (SOS) remained normal. This suggested that premature closure of the PSS was closely related to its properties of neural crest cell origin (Kolpakova-Hart et al., 2008). In contrast, in the *Dermo1*-Cre;*Pkd1* (specific knockout of *Pkd1* in mesenchymal stromal cells) mouse model, both the PSS and SOS showed developmental abnormalities. A comparison of these two models revealed that the role of *Pkd1* in different cartilage regions may be influenced by its expression pattern and cellular origin, thus affecting normal cartilage development and maturation in different ways (Kolpakova-Hart et al., 2008). In addition, *Pkd1* deletion may disrupt the normal pathway of differentiation of mesenchymal cells into chondrocytes. On day 13.5 of embryonic development, hematoxylin, and eosin staining revealed that the cranial base region of control embryos was filled with mature chondrocytes, whereas the corresponding region of mutant embryos was still filled with undifferentiated mesenchymal cells (Kolpakova-Hart et al., 2008). This finding further supports the critical role of *Pkd1* in chondrogenesis and cell differentiation. These findings provide new insights into the complex regulatory mechanisms underlying craniofacial development.

PC-1 exhibits great regulatory potential in a cellular model of premature closure of human cranial sutures (Katsianou et al., 2022). Katsianou et al. detected *Pkd1* mRNA expression using RT-PCR in primary cranial suture cells from patients with trichocephaly and polycephaly (Katsianou et al., 2021). Activation of RUNX2 by PC-1 in premature cranial suture cells is mediated by the extracellular signal-regulated kinase (ERK) signaling pathway. Specifically, RUNX2 phosphorylation levels were notably increased when cranial suture cells were treated with IgPkd1, whereas RUNX2 phosphorylation levels were markedly decreased after treatment with IgPkd1 antibody and mitogen-activated protein kinase inhibitor. This suggests that the activation of RUNX2 by PC-1 is mediated through the ERK signaling pathway (Katsianou et al., 2021). Although the ERK pathway is involved, the specific molecular mechanisms require investigation. In addition, whether PC-1 affects signaling in cranial suture cells through mechanical force signaling remains unclear; hence, this aspect is an important direction for future research.

In addition, PC-1 considerably affected the AKT/mammalian target of rapamycin (mTOR)C2 signaling pathway. In cranial suture cells with triangular and navicular head deformities, the level of phosphorylation of the AKT protein at the Ser473 site increased after PC-1 was inhibited by the IgPkd1 antibody. This suggests an increase in AKT activity. However, the expression of the effector molecules mTORC1, eukaryotic translation initiation factor 4E binding protein 1 (4EBP1), and p70S6K, was not altered by PC-1 inhibition (Katsianou et al., 2022). The mTOR complex comprises mTORC1 and mTORC2, and mTORC1’s downstream effector molecules include 4EBP1 and p70S6K (Glaviano et al., 2023). The mTORC2 molecule excites AKT by phosphorylating the Ser473 site of AKT (Glaviano et al., 2023; Gargalionis et al., 2018b). These findings suggest that PC-1 inhibition primarily affects the mTORC2 pathway (Katsianou et al., 2022). Future studies are needed to explore the specific mechanism underlying the role of PC-1 in the AKT/mTORC2 signaling pathway.

Although existing studies have revealed the role of PC-1 in ERK and AKT/mTORC2 signaling pathways, their specific signaling mechanisms need to be further elucidated. Future studies should focus on exploring the downstream signaling molecules regulated by PC-1 in these pathways, especially whether PC-1 regulates the behavior of cranial suture cells through mechano-signaling. This is expected to provide new targets and strategies for treating craniosynostosis.

## 7 Conclusions and outlook

**FIGURE 3:**
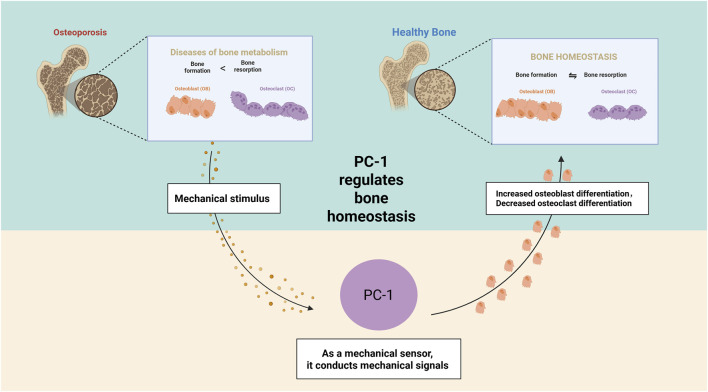
The mechanism of action of PC-1 in bone homeostasis.

In this review, the critical role of PC-1 in bone remodeling and its potential clinical applications are explored. PC-1, a complex transmembrane protein, plays a multifaceted role in osteoblasts, including mechanical force sensing, cellular signaling, and regulation of osteoblast and osteoclast functions. PC-1 directly affects the function of osteoblasts and osteoclasts, thereby regulating the balance of bone metabolism (Figure 3). The unique molecular structure of PC-1 enables it to sense mechanical force signals and transduce them into cells, affecting the activities of osteoblasts through multiple signaling pathways, including CaN/NFAT, JAK/STAT, and AKT/β-catenin. In addition, the formation of the PC-1/TAZ complex has been a breakthrough in bone metabolism research. PC-1 influences the nuclear translocation and transcriptional activity of TAZ through its interaction, playing a key role in regulating osteoblast differentiation and function. This finding provides a new perspective for understanding the molecular mechanisms of bone remodeling. Regarding clinical applications, the research results on PC-1 show great promise. PC-1 abnormalities are closely related to bone diseases, such as osteoporosis and delayed fracture healing, rendering PC-1 a promising diagnostic marker for these diseases. The development of novel therapeutic strategies targeting PC-1 is expected to lead to breakthroughs in the treatment of osteoporosis and other bone loss-related diseases.

Although the role of PC-1 in osteoblast function has been demonstrated, its detailed signaling pathways and mechanisms of interaction with other molecules need further clarification. Future studies should focus on the in-depth resolution of PC-1 signaling mechanisms, especially its specific pathways of action in mechanical force sensing and conduction, and its interactions with other molecules. Further revealing the detailed function of PC-1 in osteoclasts and its role in mechanical force signaling will provide a theoretical basis for developing new targets against skeletal diseases, such as osteoporosis. Moreover, exercise improves bone health, as does PC-1. Therefore, the relationship between exercise and PC-1-related molecular mechanisms is also an important direction for future research. By researching the effects of different exercise modalities on PC-1 and its relationship with bone remodeling, we can provide a scientific basis for applying exercise interventions in the treatment of skeletal diseases.

Finally, the development of pharmacological interventions targeting PC-1 and its associated signaling pathways will open new avenues for treating skeletal diseases. For example, the discovery of small-molecule compounds such as Zinc01442821 demonstrates their potential therapeutic promise. Future studies should further evaluate the safety and long-term effects of these interventions and explore their potential applications in different skeletal diseases.
